# Sex-dependent differences in single-leg squat kinematics and their relationship to squat depth in physically active individuals

**DOI:** 10.1038/s41598-020-76674-2

**Published:** 2020-11-11

**Authors:** Magdalena Zawadka, Jakub Smolka, Maria Skublewska-Paszkowska, Edyta Lukasik, Aleksandra Bys, Grzegorz Zielinski, Piotr Gawda

**Affiliations:** 1 Department of Sports Medicine, Faculty of Health Sciences, Medical University of Lublin, Chodzki 7, 20-093 Lublin, Poland; 2 Department of Computer Science, Faculty of Electrical Engineering and Computer Science, Lublin University of Technology, Nadbystrzycka 38D, 20-618 Lublin, Poland

**Keywords:** Risk factors, Motor control, Rehabilitation

## Abstract

The purpose of this study is to compare recreationally physically active females and males with regard to spine, pelvis and lower limb joints peak angles in each plane of motion during a single leg squat (SLS). The second aim is to investigate the relationship between kinematics and SLS depth in females and males. Fifty-eight healthy, young adults performed 5 repetitions of a single right leg squat to maximal depth while keeping their balance. Kinematic data were obtained using an optical motion capture system. At the hip, greater adduction and greater internal rotation were observed in females than in males. Females had more extended spines and less outward bended knees throughout the SLS than did men. In males, squat depth was significantly, positively correlated with the maximal angle of the ankle (r = 0.60, *p* < 0.001), the knee (r = 0.87, *p* < 0.001), the hip (r = 0.73, *p* < 0.001) and the pelvis (r = 0.40, *p* = 0.02) in the sagittal plane. A positive significant correlation was found between SLS depth and maximal angle of the knee (r = 0.88, *p* < 0.001) and the ankle (r = 0.53, *p* = 0.01) in the sagittal plane in females. Males and females used different motor strategies at all levels of the kinematic chain during SLS.

## Introduction

Functional performance tests are often used for an assessment of dynamic movement patterns that are part of more complex activity. Lower extremity kinematics and muscle activity have been documented during closed kinetic chain exercises such as jumping and landing or squatting in healthy as well as in patient populations^1–3^. These tests evaluate movement in all three planes of motion, the range of motion, strength and proprioception. One of the most commonly used functional performance tests is the single-leg squat (SLS)^4,5^. The SLS is widely used in rehabilitation, sports medicine and orthopedics^6^. It is considered a simple test, which can be used both in clinical conditions and in the investigation of large groups. It has been shown that kinematic patterns, especially those of the knee, are related to a risk of lower limb injury^7,8^. For this reason, the SLS can be used as a “screening” tool to evaluate the movement system^9,10^.

The SLS has been found valid, reliable and useful in detecting abnormal kinematics in the lower limb and trunk^9^. The SLS test is a reasonable tool to use in pre-participation sports physical examinations to assess the dynamic knee valgus and the potential risk of lower extremity injury and deficits in core strength^11,12^. Moreover, in recent years unilateral exercises, including the SLS, have become popular in strength and conditioning training. These types of exercises are included in strength programs to increase, for example, volume load^13^.

The use of the SLS test in clinical and research practice indicates the need for the study of one-leg kinematic patterns. According to some studies, reliability of the SLS test in clinical practice depends on the experience of the investigator^8^. For this reason, researchers use objective methods to evaluate the SLS, such as optical motion capture systems^14,15^. This allows for a three-dimensional analysis in all segments of the kinematic chain with high precision, high frequency and low measurement error.

Differences in kinematics between the sexes are one of the proposed risk factors for lower limb injuries in professional and recreational female athletes. Women characteristically have greater rates of knee injuries than men^16,17^. Females encounter altered frontal plane biomechanics that may predispose them to patellofemoral pain (PFP)^18^. A common mechanism by which noncontact anterior cruciate ligament (ACL) injuries occur in female athletes was described as a loss of control at the hip and pelvis, internal rotation of the femur, valgus knee and external tibial rotation on a pronated, externally rotated foot^19^. Although injuries of the ACL and PFP^20^ are a well described problem in female athletes, outcomes of studies evaluating sex differences in SLS kinematics are equivocal, especially in the analysis of pelvis and trunk motion^21–23^. There is considerable variation in how the SLS movement was performed in previous studies^24^. Moreover, the depth of squatting has not always been noted or controlled^2,22,23^.

Parameters considered as predictors of knee dysfunctions, like greater hip adduction and medial–lateral knee displacement during the SLS^25, 26^, were consistent with movement patterns observed in females^21,23^. Moreover, physically active individuals seemed to be at less risk to perform a bad SLS than non-physically active individuals^27^. Numerous studies have attempted to explain the greater risk of injuries by kinematics or muscle activity^2,22,23,28^. However, there is a lack of complex three dimensional analysis of all kinetic chain segments including spine, pelvis and lower limb joints during the SLS in reference to squat depth. Thus, the purpose of this study was to compare recreationally physically active females and males with regard to peak angles of the spine, pelvis and lower limb joints in each plane of motion during the SLS. The second aim was to investigate the relationship between kinematics and the SLS depth in females and males. We hypothesize that there should be kinematic differences between females and males at all levels of the kinematic chain during the SLS, and that kinematics would be related differently to the squatting depth with respect to gender.

## Methods

### Participants

Fifty eight healthy, young adults (35 men and 23 women) from the local student community were recruited to participate in the study. Table 1 shows the characteristics of the subjects. Participants were excluded if they had any musculoskeletal or orthopedic injury or lower limb pain. Subjects declared a high or moderate physical activity level measured using the International Physical Activity Questionnaire (IPAQ). Students gave written informed consent to participate in this study. Informed consent was obtained to publish the image in an online open access publication.

**Table 1:** Group characteristics. Statistically significant results are in bold.

Variables	Females (N = 23)		Males (N = 35)		t	p
	Mean ± SD	95%CI	Mean ± SD	95%CI		
Age [years]	20.30 ± 0.47	20.10; 20.51	21.00 ± 1.19	20.59; 21.41	**− 2.67**	**0.01**
Body length [m]	1.66 ± 0.06	1.64; 1.69	1.80 ± 0.07	1.78; 1.83	**− 8.16**	** < 0.001**
Body mass [kg]	59.31 ± 7.14	56.23; 62.40	77.15 ± 11.70	73.14; 81.17	**− 6.54**	** < 0.001**
Body Mass Index (BMI) [kg/m^2^]	21.42 ± 1.87	20.61; 22.23	23.68 ± 2.79	22.72; 24.64	**− 3.41**	**0.001**
Physical activity [MET min/week]	6059.74 ± 2536.08	4963.06; 7156.42	4677.65 ± 2762.31	3728.77; 5626.54	1.92	0.06
Sitting time at weekend [min]	678.26 ± 351.93	1372.64; 2057.80	705.71 ± 325.09	1751.20; 2287.35	**− **0.30	0.76
Sitting time during week [min]	1715.22 ± 792.21	1932.75; 2854.21	2019.29 ± 780.37	2380.86; 3069.14	**− **1.44	0.15
Total sitting time without transport [min]	2393.48 ± 1065.43	526.08; 830.45	2725.00 ± 1001.82	594.04; 817.39	**− **1.20	0.23

### Experiment design

**Figure 1:**
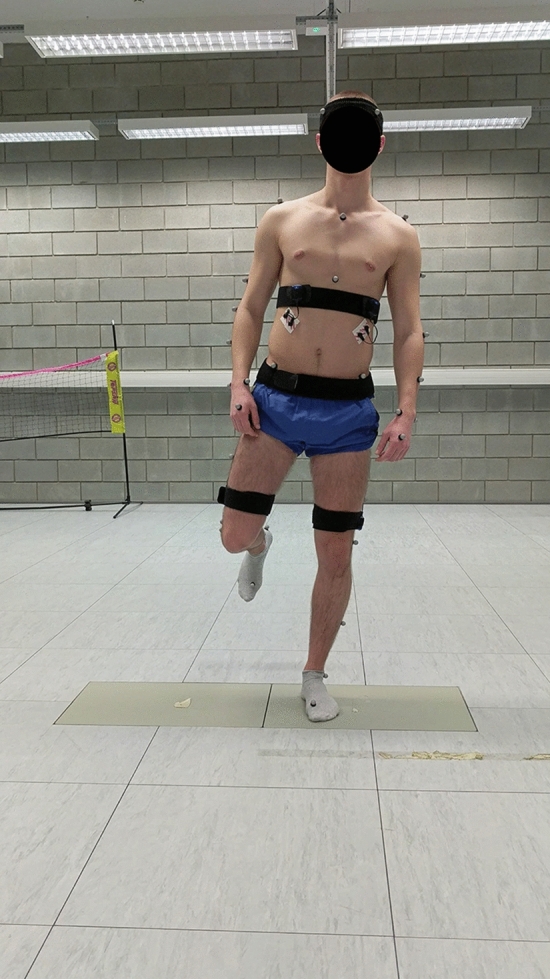
Initial position of single-leg squat.

**Figure 2:**
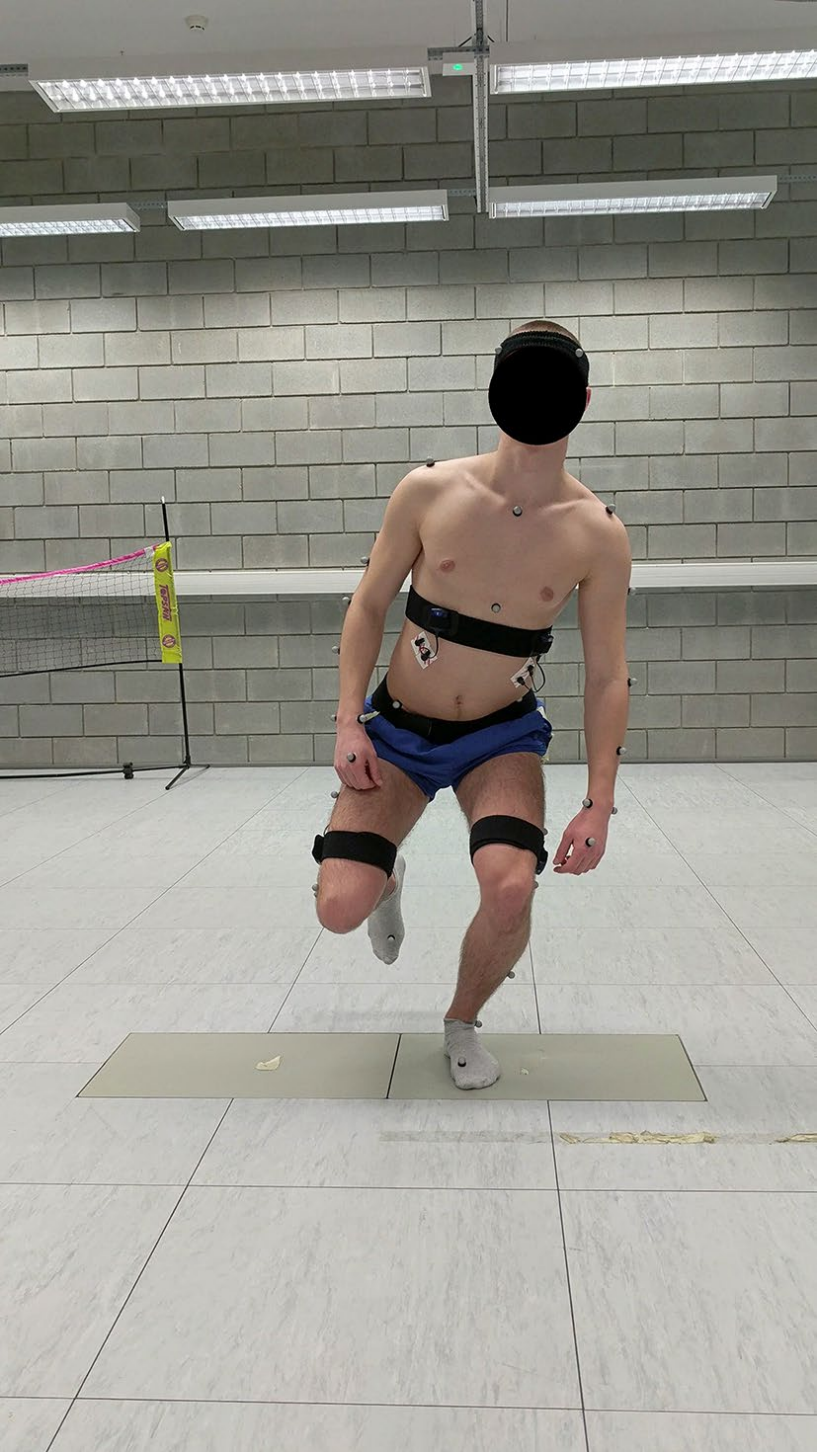
Final position of single-leg squat.

Kinematic data (100 Hz) were collected using an 8-camera, 3D motion capture system (Vicon Oxford. UK). The system registered three-dimensional trajectories of passive markers. The markers were attached to specific anatomical landmarks specified in the full-body Plug-in gait model which was defined by the system’s manufacturer. The task was demonstrated to each participant by performing a squat with the non-weight-bearing knee flexed (thigh perpendicular to the floor). Participants were instructed to look straight ahead, descend in a slow, controlled manner without losing balance and while maintaining heel contact to the ground. Before data collection a calibration trial was collected for each subject. Each participant was permitted three practice attempts before data collection. After that participants performed 5 repetitions of a single right leg squat while keeping their arms to their sides. Participants were asked to squat to maximal depth while keeping their balance, to hold the SLS position for 3 s and then to return to their initial position (Figs. 1 and 2). A trial was repeated or excluded if the performance was affected by loss of balance, incorrect non-stance leg position, or non-continuous movement. Data collection occurred in one session.

### Data analysis

An average of three middle trials (without 1st and 5th) was further analyzed. Filtered marker trajectories were used to compute a three-dimensional segment (trunk and pelvis) and joint kinematics using the BodyBuilder modelling software (Vicon; Oxford Metrics). Results were compared between females and males in three dimensions according to the maximal and minimal angle reached during the descent phase of motion. The angles were calculated using the Euler angle method in a flexion/extension, abduction/adduction and internal/external rotation sequence. Positive values mean: (1) for the spine : flexion, lateral flexion towards the stance leg, rotation to the side opposite to the stance leg; (2) for the pelvis : anterior tilt, obliquity towards the stance leg, rotation to the side opposite to the stance leg; (3) for the hip : flexion, adduction and external rotation; (4) for the knee : flexion, varus (outward bend) and internal rotation; (5) for the ankle : dorsiflexion, inversion and internal rotation^29^. Squat depth was defined as the difference in S2 marker height (second sacral spine process) according to the following equation:

$$\Delta h = \frac{{h_{max} - h_{min} }}{{h_{max} }} \times 100{\text{\% }}$$

$\Delta h$-squat depth, ${h}_{max}$-the highest position of S2 marker, ${h}_{min}$-the lowest position of S2 marker.

The squat depth was expressed as a percentage of leg length measured vertically based on the S2 marker position. The leg length was defined as the distance from the ground to the S2 marker during the initial position of the single-leg squat. The leg length in our study represents the total limb length including hip joint.

### Statistical analysis

Data analysis was conducted using the Statistica software (ver. 13.1). The Shapiro–Wilk test revealed that the data was distributed normally. Therefore, to address the primary purpose of the study, independent t-tests were utilized to compare females and males in regard to joint peak angles (minimal and maximal) in each plane of motion. Effect sizes were determined using the Cohen d coefficient. The Pearson correlation was used to examine the relationships between (1) the angles of the lumbar spine, pelvis, hip, knee and ankle achieved during the squatting; (2) the squat depth and anthropometric variables (body length, body mass and BMI) and the squat depth. Statistical significance was accepted at *p* < 0.05 with all the outcome measures reported as mean, standard deviation (SD) and 95% confidence interval (95% CI). The graphs were prepared using Microsoft Office Excel.

Intraclass correlation coefficients (ICCs) were calculated to assess the reliability of the repeated measures. Interpretation of ICC values was made according to the following scale: < 0.40 represented poor reliability, 0.40–0.70 fair reliability, 0.70–0.90 good reliability and > 0.9 excellent reliability^30^. Reliability close to one indicates that the measurement is consistent with no big change in trait size between measurements.

### Ethics approval

This study was performed in line with the principles of the Helsinki Declaration. Approval was granted by the Medical University of Lublin Bio Ethics Committee (approval number KE-0254/322/2018).

## Results

ICCs of squat descent repeated measures showed good reliability (ICC = 0.86). 6.5% of ICC values showed fair reliability (ICC = 0.40–0.70), 41.9% of ICC values showed good reliability (ICC = 0.70–0.90) and 51.6% of ICC values showed excellent reliability (ICC > 0.9) of the repeated measures. The ICCs for the dependent variables are included in Table 2.

**Table 2:** The ICC values for the dependent variables.

Angle	Ankle	Knee	Hip	Pelvis	Spine
Sagittal max	0.91	0.91	0.94	0.93	0.97
Sagittal min	0.85	0.70	0.78	0.88	0.96
Frontal max	0.95	0.96	0.87	0.80	0.82
Frontal min	0.96	0.86	0.88	0.83	0.76
Transverse max	0.96	0.97	0.98	0.68	0.87
Transverse min	0.95	0.93	0.92	0.81	0.91

### Kinematics

**Figure 3:**
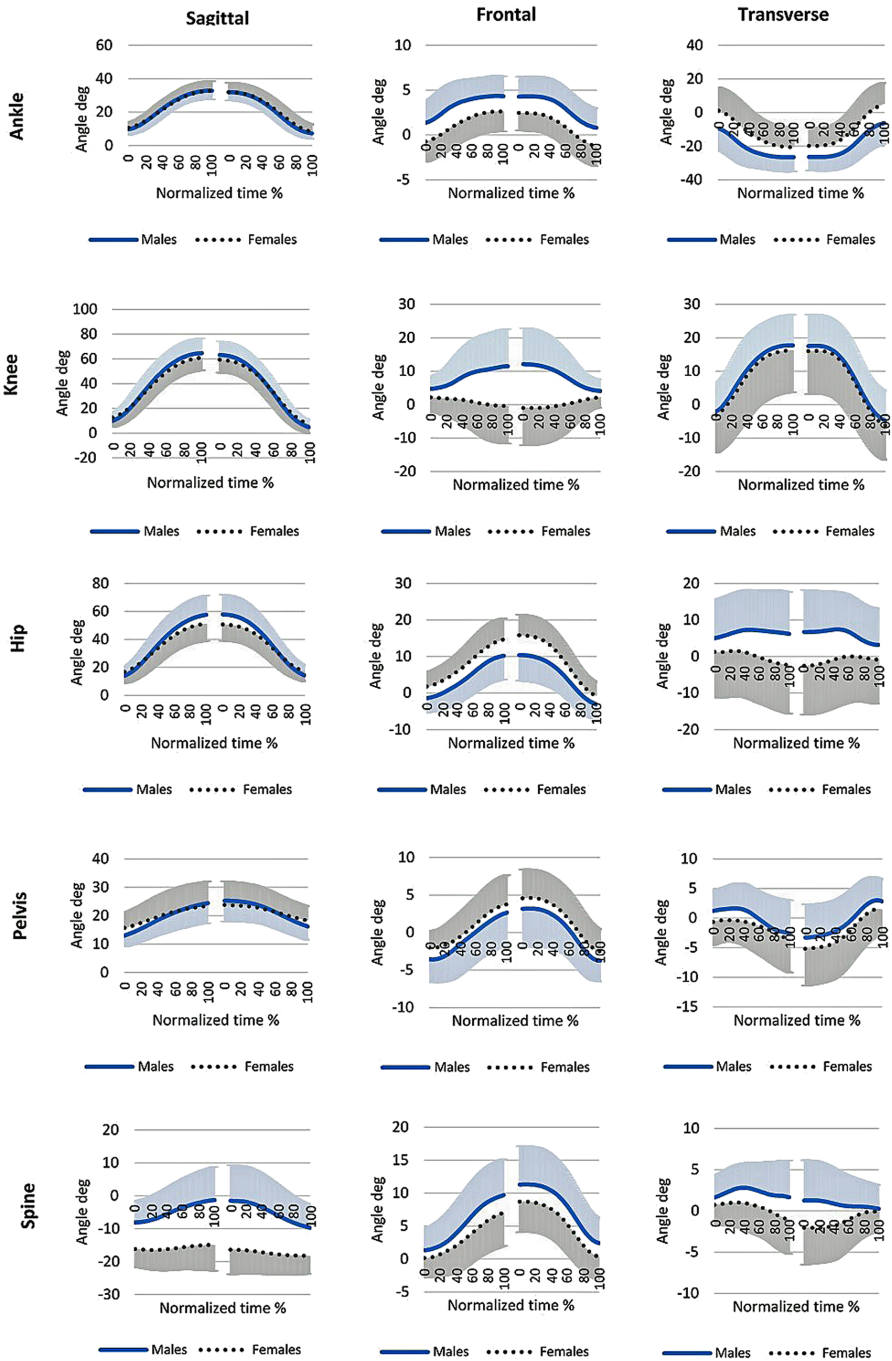
3D motion of each segment during SLS. Shaded error bands (single-sided) represent one standard deviation.

Statistical analysis revealed a number of differences between the sexes (Table 3). At the ankle, males showed greater minimal and maximal angles in the frontal plane (1.28° vs. − 0.79°, *p* = 0.001; and 4.77° vs. 2.80°, *p* = 0.003) and lower angles in the transverse plane (− 8.87° vs. 1.44°, *p* = 0.004; and − 28.59° vs. − 21.64°, *p* = 0.01, for maximal and minimal angle respectively) than females. These angles indicate a more inverted and externally rotated foot in males. At the knee, sex differences were observed only in the frontal plane, where men exhibited a more outward bended knee (varus) than women (13.14° vs. 4.93° and 2.69° vs. − 3.43°, for maximal and minimal angle respectively, both *p* < 0.001). At the hip, greater adduction (14.92° vs. 10.43°, *p* = 0.006 and 1.78° vs. − 1.80°, *p* < 0.001 for maximal and minimal angle in the frontal plane respectively), less external rotation (3.32° vs. 10.07°, *p* = 0.02) and greater internal rotation (− 4.00° vs. 2.6°, *p* = 0.04) were observed in females than in males. At the pelvis, the minimal angle of pelvic anterior tilt was greater in females than in males (15.56° vs. 12.95°, *p* = 0.04). Males, for instance, demonstrated a greater minimal obliquity of the pelvis (− 4.04° vs. − 2.36°, *p* = 0.02). There was a significant difference in spine motion in the sagittal plane. Females had a more extended spine throughout the SLS (− 13.43° vs. − 0.77°, and − 18.18° vs. − 8.60°, *p* < 0.001 for maximal and minimal angle in the sagittal plane respectively) and a less rotated spine to the side opposite to the stance leg than males (1.78° vs. 3.80°, *p* = 0.01). Moreover, males demonstrated a greater rotation of the pelvis to the side opposite to the stance leg (2.95° vs. 1.03°) and a greater spine lateral flexion towards the stance leg than females (10.02° vs. 7.37°). However, these results were on the border of significance (*p* = 0.05). Figure 3 demonstrates the angles in all three planes in normalized time (0–100% for the descent and ascent phase separately).

**Table 3:** Comparison of kinematics parameters between females and males. Statistically significant results are in bold. Max, min denote the maximal and the minimal angles reached during the descent phase of motion.

Variables	Females (N = 23)				Males (N = 35)				t	p	Effect size Cohen’s d
	Mean	SD	**− **95%Cl	+ 95%Cl	Mean	SD	**− **95%Cl	+ 95%Cl			
**Ankle**											
Sagittal max [°]	33.07	5.61	30.64	35.49	33.25	4.90	31.57	34.93	**− **0.13	0.90	0.03
Sagittal min [°]	10.57	2.63	9.43	11.71	9.65	3.12	8.57	10.72	1.17	0.25	0.32
Frontal max [°]	2.80	2.26	1.83	3.78	4.77	2.40	3.95	5.60	**− **3.13	**0.003**	0.85
Frontal min [°]	**− **0.79	2.25	**− **1.76	0.19	1.28	2.29	0.49	2.07	**− **3.38	**0.001**	0.91
Transverse max [°]	1.44	13.67	**− **4.47	7.35	**− **8.87	12.40	**− **13.12	**− **4.61	2.97	**0.004**	0.79
Transverse min [°]	**− **21.64	11.75	**− **26.72	**− **16.56	**− **28.59	8.43	**− **31.49	**− **25.70	2.63	**0.01**	0.69
**Knee**											
Sagittal max [°]	60.95	9.57	56.81	65.09	64.92	11.76	60.88	68.96	**− **1.35	0.18	0.37
Sagittal min [°]	12.48	7.21	9.36	15.60	9.92	4.86	8.26	11.59	1.62	0.11	0.42
Frontal max [°]	4.93	8.24	1.37	8.50	13.40	8.77	10.39	16.41	**− **3.68	** < 0.001**	1.00
Frontal min [°]	**− **3.43	7.18	**− **6.54	**− **0.33	2.69	5.85	0.68	4.70	**− **3.56	** < 0.001**	0.94
Transverse max [°]	16.73	12.70	11.24	22.22	18.42	8.88	15.37	21.47	**− **0.60	0.55	0.16
Transverse min [°]	**− **3.35	11.22	**− **8.20	1.50	**− **2.44	8.06	**− **5.21	0.32	**− **0.36	0.72	0.09
**Hip**											
Sagittal max [°]	51.31	11.94	46.15	56.47	57.68	13.55	53.03	62.34	**− **1.84	0.07	0.50
Sagittal min [°]	16.83	7.78	13.47	20.19	13.96	5.22	12.17	15.76	1.68	0.10	0.44
Frontal max [°]	14.92	5.54	12.52	17.32	10.43	6.01	8.37	12.50	2.87	**0.006**	0.78
Frontal min [°]	1.78	3.73	0.17	3.40	**− **1.80	3.84	**− **3.11	**− **0.48	3.51	** < 0.001**	0.95
Transverse max [°]	3.32	12.06	**− **1.90	8.53	10.07	9.68	6.74	13.40	**− **2.36	**0.02**	0.62
Transverse min [°]	**− **4.00	13.42	**− **9.80	1.80	2.66	11.07	**− **1.14	6.46	**− **2.06	**0.04**	0.54
**Pelvis**											
Sagittal max [°]	23.84	8.29	20.26	27.43	24.61	6.70	22.31	26.91	**− **0.39	0.70	0.10
Sagittal min [°]	15.56	5.40	13.23	17.90	12.95	3.85	11.63	14.28	2.15	**0.04**	0.56
Frontal max [°]	3.92	3.68	2.33	5.52	2.88	4.15	1.46	4.31	0.97	0.33	0.27
Frontal min [°]	**− **2.36	2.25	**− **3.33	**− **1.39	**− **4.04	2.89	**− **5.03	**− **3.04	2.35	**0.02**	0.65
Transverse max [°]	1.03	3.72	**− **0.58	2.64	2.95	3.55	1.73	4.16	**− **1.98	0.05	0.53
Transverse min [°]	**− **4.46	4.77	**− **6.53	**− **2.40	**− **3.61	4.75	**− **5.24	**− **1.98	**− **0.67	0.51	0.18
**Spine**											
Sagittal max [°]	**− **13.43	6.76	**− **16.35	**− **10.51	**− **0.77	9.50	**− **4.04	2.49	**− **5.53	** < 0.001**	1.56
Sagittal min [°]	**− **18.18	6.57	**− **21.02	**− **15.34	**− **8.60	6.57	**− **10.85	**− **6.34	**− **5.44	** < 0.001**	1.46
Frontal max [°]	7.37	4.63	5.37	9.37	10.02	5.03	8.29	11.75	**− **2.03	0.05	0.55
Frontal min [°]	**− **0.32	3.12	**− **1.67	1.03	0.86	3.17	**− **0.23	1.95	**− **1.39	0.17	0.38
Transverse max [°]	1.78	2.76	0.58	2.97	3.80	2.99	2.77	4.83	**− **2.60	**0.01**	0.70
Transverse min [°]	**− **1.61	3.84	**− **3.27	0.06	0.06	3.64	**− **1.19	1.31	**− **1.67	0.10	0.45
**Depth [%leg length]**											
SLS depth	13.10%	3.20	11.70%	14.50%	15.00%	4.40	13.50%	16.50%	− 1.81	0.08	0.50

### Relationship between kinematics and SLS depth

A positive significant correlation was found between SLS depth and the maximal angle of the knee (r = 0.88, *p* < 0.001) and the ankle (r = 0.53, *p* = 0.01) in the sagittal plane in females. The minimal angle of ankle dorsiflexion was negatively correlated to squat depth in women (r = − 0.50, *p* = 0.01). In males the squat depth was significantly, positively correlated with the maximal angle of ankle (r = 0.60, *p* < 0.001), knee (r = 0.87, *p* < 0.001), hip (r = 0.73, *p* < 0.001) and pelvis (r = 0.40, *p* = 0.02) in the sagittal plane and the maximal angle of knee (r = 0.43, *p* = 0.01) in the transverse plane (Table 4).

**Table 4:** Results of Pearson correlation for descent and kinematics parameters in females and males. Statistically significant results are in bold. Max, min denote the maximal and the minimal angles reached during the descent phase of motion.

Variables	Females (N = 23)				Males (N = 35)			
	r(X.Y)	r2	**t**	**p**	r(X.Y)	r2	**t**	**p**
**Ankle**								
Sagittal max [°]	0.53	0.28	2.83	**0.01**	0.60	0.35	4.26	** < 0.001**
Sagittal min [°]	**− **0.50	0.25	**− **2.67	**0.01**	**− **0.20	0.04	**− **1.17	0.25
Frontal max [°]	0.00	0.00	0.01	0.99	**− **0.15	0.02	**− **0.88	0.38
Frontal min [°]	**− **0.02	0.00	**− **0.11	0.91	**− **0.25	0.06	**− **1.51	0.14
Transverse max [°]	0.13	0.02	0.62	0.54	0.24	0.06	1.42	0.16
Transverse min [°]	**− **0.01	0.00	**− **0.03	0.97	0.13	0.02	0.75	0.46
**Knee**								
Sagittal max [°]	0.88	0.77	8.38	** < 0.001**	0.93	0.87	14.80	** < 0.001**
Sagittal min [°]	**− **0.32	0.10	**− **1.54	0.14	**− **0.23	0.05	**− **1.38	0.18
Frontal max [°]	**− **0.06	0.00	**− **0.28	0.78	**− **0.02	0.00	**− **0.09	0.93
Frontal min [°]	**− **0.04	0.00	**− **0.17	0.87	**− **0.14	0.02	**− **0.81	0.43
Transverse max [°]	0.18	0.03	0.82	0.42	0.43	0.18	2.72	**0.01**
Transverse min [°]	**− **0.01	0.00	**− **0.03	0.97	0.12	0.01	0.71	0.48
**Hip**								
Sagittal max [°]	0.19	0.03	0.87	0.40	0.73	0.53	6.14	** < 0.001**
Sagittal min [°]	**− **0.27	0.08	**− **1.31	0.20	**− **0.17	0.03	**− **0.99	0.33
Frontal max [°]	**− **0.17	0.03	**− **0.80	0.43	0.29	0.08	1.71	0.10
Frontal min [°]	**− **0.04	0.00	**− **0.17	0.87	**− **0.03	0.00	**− **0.15	0.88
Transverse max [°]	0.09	0.01	0.42	0.68	**− **0.10	0.01	**− **0.55	0.59
Transverse min [°]	0.04	0.00	0.17	0.87	**− **0.14	0.02	**− **0.83	0.42
**Pelvis**								
Sagittal max [°]	**− **0.21	0.04	**− **0.96	0.35	0.40	0.16	2.47	**0.02**
Sagittal min [°]	**− **0.03	0.00	**− **0.16	0.87	0.02	0.00	0.13	0.90
Frontal max [°]	**− **0.38	0.15	**− **1.91	0.07	0.13	0.02	0.78	0.44
Frontal min [°]	**− **0.09	0.01	**− **0.40	0.69	**− **0.04	0.00	**− **0.22	0.82
Transverse max [°]	**− **0.08	0.01	**− **0.36	0.72	**− **0.29	0.09	**− **1.75	0.09
Transverse min [°]	0.09	0.01	0.42	0.68	**− **0.29	0.09	**− **1.77	0.09
**Spine**								
Sagittal max [°]	**− **0.01	0.00	**− **0.04	0.97	0.24	0.06	1.40	0.17
Sagittal min [°]	0.00	0.00	**− **0.02	0.99	**− **0.03	0.00	**− **0.18	0.86
Frontal max [°]	**− **0.32	0.10	**− **1.54	0.14	0.13	0.02	0.77	0.45
Frontal min [°]	**− **0.01	0.00	**− **0.03	0.98	**− **0.04	0.00	**− **0.24	0.81
Transverse max [°]	0.11	0.01	0.49	0.63	**− **0.16	0.02	**− **0.91	0.37
Transverse min [°]	0.20	0.04	0.92	0.37	**− **0.31	0.10	**− **1.86	0.07

Moreover, correlation of the squat depth and anthropometric measurements was performed (Table 5). The analysis indicated that there was no statistically significant relationship between body mass, height, Body Mass Index (BMI) and squat depth.

**Table 5:** Results of Pearson correlation for squat depth (% leg length) and body mass, body length and BMI.

Variables	All participants (N = 58)				Females (N = 23)				Males (N = 35)			
	r(X,Y)	r2	**t**	**p**	r(X,Y)	r2	**t**	**p**	r(X,Y)	r2	**t**	**p**
Body mass [kg]	0.01	0.00	0.08	0.93	**− **0.19	0.04	**− **0.90	0.38	**− **0.20	0.04	**− **1.16	0.25
Body length [m]	0.01	0.00	0.08	0.94	**− **0.32	0.10	**− **1.52	0.14	**− **0.22	0.05	**− **1.29	0.20
BMI [kg/m^2^]	0.02	0.00	0.13	0.90	0.03	0.00	0.14	0.89	**− **0.13	0.02	**− **0.75	0.46

## Discussion

The purpose of this study was to compare recreationally physically active females and males with regard to the spine, pelvis and peak angles of the lower limb joints in each plane of motion during the SLS and to investigate the relationship between kinematics and SLS depth. The study results demonstrate that males and females use different motor strategies at all levels of the kinematic chain. The differences are noted especially in the frontal and the transverse planes in the lower limbs’ joints and in the sagittal plane of the spine.

According to Zeller et al., females demonstrated significantly more ankle dorsiflexion, ankle pronation, hip adduction, flexion and external rotation, and less trunk lateral flexion than men in SLS^22^. In the present study, differences in ankle motion in the transverse and frontal planes occurred in a comparison of males and females. In accordance with the Zeller findings, females demonstrated smaller inversion and smaller external rotation of the foot than men in our study. Greater foot external rotation observed in this study in males is probably related to greater knee varus. Previously it had been noticed by researchers that factors of foot mobility should be included in the clinical treatment of knee-related disorders^31,32^. According to our findings, examiners of the SLS should also take into consideration sex-dependent differences of ankle and foot motion, not only knee and hip.

When performing the SLS, females, compared with males, show a characteristic kinematic profile of the lower limb observed previously in the literature with higher hip internal rotation, hip adduction and knee valgus^23,33,34^. A similar study to the one presented here was conducted by Weeks et al., who examined 30 men (25.6 ± 4.8 years) and 30 women (25.1 ± 3.8 years)^21^. Both studies were characterized by similarly sized study groups, their ages and research methods. In the research of Weeks et al., the peak internal hip rotation (− 1.8° ± 5.7° vs.. 3.0° ± 7.3°) and the hip adduction range (11.7° ± 4.8° vs.. 18.3° ± 6.7°) seemed to be smaller for males than for females^21^. The above sex differences were also observed in the authors’ study. The hip rotation range in females in our study was between − 4.00° and 3.32°, while males kept only an external rotation in the range of 2.66°–10.07° throughout the SLS. The mean peak hip adduction was 14.92° ± 5.54° in females and 10.43° ± 6.01° in males. Our findings are in opposition to the conclusion described by Zeller et al., who noted that female subjects tended to rotate their pelvis away from the stance leg and had a more externally rotated hip during the SLS^22^. In our study males rotated their pelvis and spine to a greater degree away from the stance leg and, as a result, they had greater hip external rotation. Previous studies report that increased hip adduction and hip internal rotation have been associated with higher levels of pain and reduced function in patients with PFP^35,36^. However, it remains unclear how the differences observed in this study can be related to pain and injury risk in healthy participants. Given this, long term prospective studies can explain if these SLS kinematics translate into injuries for males and females in different ways.

In the current study males have a more outward bended knee (varus) than females (13.14° vs. 4.93° and 2.69° vs. − 3.43°, for maximal and minimal angle in the frontal plane respectively). Apparent knee valgus has been considered by clinicians as one of the criterions describing “poor” SLS performance^37,38^. Despite statistically significant differences between males and females, the mean knee valgus obtained by females (− 3.43°) is small and should be considered as a normal, rather than pathological, value^39,40^. Differences in knee kinematics in the frontal plane are similar to those reported by Graci et. al in a group of healthy adults who were not physically active on a regular basis and were about seven years older than the participants in this study^23^.

Statistically significant differences in pelvis motion were observed between males and females in the present study in the frontal and sagittal planes. They are inconsistent with the previous studies of Weeks et al. and Graci et al., who found statistically significant differences between males and females only in pelvic rotations^21,23^. During the SLS test, Graci et al. observed that females flexed their torsos less than men and, more than males, turned their torsos and pelvises toward the stance limb^23^. In the current study females had more extended spines throughout the SLS and less rotated spines to the side opposite to the stance leg than males did. Moreover, males demonstrated a greater rotation of the pelvis to the side opposite to the stance leg and a greater lateral trunk flexion towards the stance leg than females did. The clinical significance of the observed differences in pelvic rotation, trunk lateral flexion and rotation warrant further investigation with respect to injury incidence. The observed differences between our findings in this study and a previous study of Graci et al. could be caused by the smaller sample size in Graci’s investigation where only 9 women and 10 men were compared. Also, in the study of Graci et al. measurements were taken at 45° of knee flexion and at peak knee flexion, but the mean peak flexions were 69.77° ± 7.27° for females and 76.43° ± 10.15° for males, which means squat depth was greater than in the current study. Similar knee flexions during the maximal SLS to the results of the present study were observed by Dwyer et al. who reported an average of 66.8° in males and of 60.0° in females^2^. However, Dwyer et al. did not investigate the trunk motion and did not find any statistically significant differences between sexes in the frontal and transverse motions of the hip and the knee, suggesting that significant alternations in these planes are related to greater depths, exceeding 60 degrees.

It was observed in this study that females had a more upright posture during squatting than males did. This observation is consistent with the previous studies of Graci et al.^23^. However, Weeks et al. did not report any differences between the sexes in the trunk motion. The result obtained in this study may be explained by the squatting procedure. In Weeks’ study participants squat down to achieve knee flexion of between 75° and 85°, and in the present study the SLS is performed to the preferred maximal depth without imposition of knee flexion (which was on average smaller than in Weeks’ study and achieved 60.95° in females and 64.92° in males)^21^. The high values of knee flexion related to greater depth in Weeks’ investigation are probably causes of a more flexed trunk in both females and males. However, in the squat depth range which we investigated, the trunk mobility in the sagittal plane was not related to squat depth. According to Powers et al., a more upright posture in females can be explained by a lack of hip extensor force to control the forward mass center shift during the descent phase. As a result, women have to rely on the quadriceps muscle, which indicates a strategy that could put ACL at a risk of injury^41^. Single-leg squats performed with a moderate forward trunk lean can minimize anterior cruciate ligament loads. The increase in forward trunk lean can also result in a concomitant increase in hip flexion which would shorten the rectus femoris muscle and lengthen the hamstrings. This mechanism may affect the thigh muscle forces^42^. According to research by Mendiguchia et al., females show a greater lateral displacement of the torso, and increased hip adduction and internal rotation during sports maneuvers when compared to males^43^. The results of the authors’ research were convergent except for the lateral displacement of the torso which was greater in the male group.

An analysis of the relationship between kinematics and SLS depth shows differences in males and females, which is consistent with the stated hypothesis. A possible explanation of some opposing results of this and previous studies mentioned above can be in the way that the SLS is executed. Khuu et al. demonstrated that execution of the SLS can significantly affects kinematics^44,45^. In the literature the SLS execution is described in a variety of ways. Hands can be placed across the chest^46^, at the sides^47^, out to the sides^34^ or placed on the pelvis^21^. There are also large variations in the manner in which the non-stance leg is positioned during the SLS. It can be extended out front^44^, held in line with the ankle of the stance leg^44^, with the knee flexed 90° while maintaining a vertical thigh position (behind the weight-bearing leg)^34^. Some studies did not explicitly state the position of the non-stance leg^35,47^. Considerable variation in how the SLS movement is performed in many cases do not allow comparison between studies^24^.

Another explanation of incompatible results of this and previous studies might be the lack of depth control in squatting. In this study, as well as in previous investigations, participants were instructed to squat down as far as possible. The depth of squatting was not imposed, as we thought that this better represented a clinical setting in which normal inter-participant variability would exist. However, to the best of the authors’ knowledge, this study is the first one to analyze the relationship between SLS depth and three-dimensional kinematics. This study shows that, in spite of the fact that the average squat depths in females and males were similar, SLS depth was related to a greater number of kinematics parameters in males than in females. In females the depth was related only to the ankle and the knee kinematics in the sagittal plane. This indicates that women rely mostly on these joints during their SLS descent. Males show a more complex motion, involving the hip, pelvis and transverse motion of the knee involved in the SLS descent. At the same time, SLS kinematics in females seem to be less predictable because only a few parameters are related to squat depth and others occur more independently. Depth can be an important factor affecting motion kinematics and muscle activity^2^. Thus, clinical evaluation of the SLS should include an analysis of depth and kinematics to understand how SLS depth can alter kinematics in males and females. However, our study indicates that SLS depth is poorly related to frontal and transverse plane kinematics, thus differences in these planes between the sexes can be observed even when SLS depth is small. Based on these findings, it seems unlikely that women lack control of the hip and knee stabilizing muscles to maintain proper frontal-plane and transverse-plane motion when SLS depth increases, as was previously suggested^2^.

Comparison of anthropometric variables (body length, body mass, BMI) showed significant differences between males and females. However, correlation of these values with squat depth indicate that there was no statistically significant relationship between them. A previous study by Mckean and Burkett reported that body height can be related to kinematic parameters in males and females during the two-leg back squat^48^. Thus, anthropometric parameters seem to be linked to SLS depth affecting kinematics. Nevertheless, in our study squat depth was related to kinematics, but not to height, body mass and BMI, neither in males nor in females.

This study has some limitations. First, we acknowledge a lack of older participants and those not physically active. Such a comparison between young and older persons and between more and less physically active ones might shed some new light on the topic of evaluating SLS kinematics. Second, in this study we used no limb dominance evaluation, which may have some influence on motor control^49^. Nevertheless, our study is the first detailed three-dimensional analysis of the SLS in relation to squat depth.

In conclusion, during the SLS males and females demonstrated similar lower limb motion in the sagittal plane, but different spine motion. Males and females showed different kinematics during the SLS in the frontal and transverse planes in all segments of the kinematic chain. Different kinematics of the trunk in the sagittal plane and lower limbs in the frontal and transverse planes of males and females should be considered by clinicians and coaches during an examination of SLS performance. Squat depth should be considered as a factor differently related, in men and women, to the motion of the kinematic chain during the SLS.

## Data Availability

The datasets generated during and/or analyzed during the current study are available from the corresponding author on reasonable request.

## References

[1] Alahmari A, Herrington L, Jones R (2020). Concurrent validity of two-dimensional video analysis of lower-extremity frontal plane of movement during multidirectional single-leg landing. Phys. Ther. Sport.

[2] Dwyer MK, Boudreau SN, Mattacola CG, Uhl TL, Lattermann C (2010). Comparison of lower extremity kinematics and hip muscle activation during rehabilitation tasks between sexes. J. Athl. Train..

[3] Gawda P (2019). Bioelectrical activity of vastus medialis and rectus femoris muscles in recreational runners with anterior knee pain. J. Hum. Kinet..

[4] McGovern RP, Martin RL, Christoforetti JJ, Kivlan BR (2018). Evidence-based procedures for performing the single leg squat and step-down tests in evaluation of non-arthritic hip pain: a literature review. Int. J. Sports Phys. Ther..

[5] Marshall AN, Hertel J, Hart JM, Russell S, Saliba SA (2020). Visual biofeedback and changes in lower extremity kinematics in individuals with medial knee displacement. J. Athl. Train..

[6] Kianifar R, Lee A, Raina S, Kulić D (2017). Automated assessment of dynamic knee valgus and risk of knee injury during the single leg squat. IEEE J. Transl. Eng. Health Med..

[7] Mohr M, von Tscharner V, Whittaker JL, Emery CA, Nigg BM (2019). Quadriceps-hamstrings intermuscular coherence during single-leg squatting 3–12 years following a youth sport-related knee injury. Hum. Mov. Sci..

[8] Weeks BK, Carty CP, Horan SA (2012). Kinematic predictors of single-leg squat performance: a comparison of experienced physiotherapists and student physiotherapists. BMC Musculoskelet. Disord..

[9] Lewis CL, Foch E, Luko MM, Loverro KL, Khuu A (2015). Differences in lower extremity and trunk kinematics between single leg squat and step down tasks. PLoS ONE.

[10] Wasserberger K, Barfield J, Anz A, Andrews J, Oliver G (2019). Using the single leg squat as an assessment of stride leg knee mechanics in adolescent baseball pitchers. J. Sci. Med. Sport.

[11] Okada T, Huxel KC, Nesser TW (2011). Relationship between core stability, functional movement, and performance. J. Strength Cond. Res..

[12] Munro A, Herrington L, Comfort P (2017). The relationship between 2-dimensional knee-valgus angles during single-leg squat, single-leg-land, and drop-jump screening tests. J. Sport Rehabil..

[13] Eliassen W, Saeterbakken AH, van den Tillaar R (2018). Comparison of bilateral and unilateral squat exercises on barbell kinematics and muscle activation. Int. J. Sports Phys. Ther..

[14] Windolf M, Götzen N, Morlock M (2008). Systematic accuracy and precision analysis of video motion capturing systems–exemplified on the Vicon-460 system. J. Biomech..

[15] Tsushima H, Morris ME, McGinley J (2003). Test-retest reliability and inter-tester reliability of kinematic data from a three-dimensional gait analysis system. J. Jpn. Phys. Ther. Assoc..

[16] Prodromos CC, Han Y, Rogowski J, Joyce B, Shi K (2007). A meta-analysis of the incidence of anterior cruciate ligament tears as a function of gender, sport, and a knee injury-reduction regimen. Arthrosc. J. Arthrosc. Relat. Surg. Off. Publ. Arthrosc. Assoc. N. Am. Int. Arthrosc. Assoc..

[17] Francis P, Whatman C, Sheerin K, Hume P, Johnson MI (2019). The proportion of lower limb running injuries by gender, anatomical location and specific pathology: a systematic review. J. Sports Sci. Med..

[18] Nakagawa TH, Moriya ÉTU, Maciel CD, Serrão AFV (2012). Frontal plane biomechanics in males and females with and without patellofemoral pain. Med. Sci. Sports Exerc..

[19] Ireland ML (1999). Anterior cruciate ligament injury in female athletes: epidemiology. J. Athl. Train..

[20] Boling M (2010). Gender differences in the incidence and prevalence of patellofemoral pain syndrome. Scand. J. Med. Sci. Sports.

[21] Weeks BK, Carty CP, Horan SA (2015). Effect of sex and fatigue on single leg squat kinematics in healthy young adults. BMC Musculoskelet. Disord..

[22] Zeller BL, McCrory JL, Kibler WB, Uhl TL (2003). Differences in kinematics and electromyographic activity between men and women during the single-legged squat. Am. J. Sports Med..

[23] Graci V, Van Dillen LR, Salsich GB (2012). Gender differences in trunk, pelvis and lower limb kinematics during a single leg squat. Gait Posture.

[24] Warner MB (2019). A systematic review of the discriminating biomechanical parameters during the single leg squat. Phys. Ther. Sport.

[25] Shirey M (2012). The influence of core musculature engagement on hip and knee kinematics in women during a single leg squat. Int. J. Sports Phys. Ther..

[26] Horan SA, Watson SL, Carty CP, Sartori M, Weeks BK (2014). Lower-limb kinematics of single-leg squat performance in young adults. Physiother. Can..

[27] Gianola S, Castellini G, Stucovitz E, Nardo A, Banfi G (2017). Single leg squat performance in physically and non-physically active individuals: a cross-sectional study. BMC Musculoskelet. Disord..

[28] Brund RBK (2018). The association between eccentric hip abduction strength and hip and knee angular movements in recreational male runners: an explorative study. Scand. J. Med. Sci. Sports.

[29] Svoboda Z, Janura M, Kutilek P, Janurova E (2016). Relationships between movements of the lower limb joints and the pelvis in open and closed kinematic chains during a gait cycle. J. Hum. Kinet..

[30] McKean MR, Dunn PK, Burkett BJ (2010). The lumbar and sacrum movement pattern during the back squat exercise. J. Strength Cond. Res..

[31] Wyndow N (2016). The relationship of foot and ankle mobility to the frontal plane projection angle in asymptomatic adults. J. Foot Ankle Res..

[32] Kagaya Y, Fujii Y, Nishizono H (2015). Association between hip abductor function, rear-foot dynamic alignment, and dynamic knee valgus during single-leg squats and drop landings. J. Sport Health Sci..

[33] Yamazaki J, Muneta T, Ju YJ, Sekiya I (2010). Differences in kinematics of single leg squatting between anterior cruciate ligament-injured patients and healthy controls. Knee Surg. Sports Traumatol . Arthrosc. Off. J. ESSKA.

[34] Graci V, Salsich GB (2015). Trunk and lower extremity segment kinematics and their relationship to pain following movement instruction during a single-leg squat in females with dynamic knee valgus and patellofemoral pain. J. Sci. Med. Sport.

[35] Nakagawa TH, Moriya ETU, Maciel CD, Serrão FV (2012). Trunk, pelvis, hip, and knee kinematics, hip strength, and gluteal muscle activation during a single-leg squat in males and females with and without patellofemoral pain syndrome. J. Orthop. Sports Phys. Ther..

[36] Nakagawa TH, Serrão FV, Maciel CD, Powers CM (2013). Hip and knee kinematics are associated with pain and self-reported functional status in males and females with patellofemoral pain. Int. J. Sports Med..

[37] Hollman JH, Galardi CM, Lin I-H, Voth BC, Whitmarsh CL (2014). Frontal and transverse plane hip kinematics and gluteus maximus recruitment correlate with frontal plane knee kinematics during single-leg squat tests in women. Clin. Biomech. Bristol Avon.

[38] Crossley KM, Zhang W-J, Schache AG, Bryant A, Cowan SM (2011). Performance on the single-leg squat task indicates hip abductor muscle function. Am. J. Sports Med..

[39] Mendonça LD (2011). Normative values of dynamic knee valgus during single leg squat in basketball and volleyball athletes. Br. J. Sports Med..

[40] Gilmer GG, Gascon SS, Oliver GD (2018). Classification of lumbopelvic-hip complex instability on kinematics amongst female team handball athletes. J. Sci. Med. Sport.

[41] Powers CM (2010). The influence of abnormal hip mechanics on knee injury: a biomechanical perspective. J. Orthop. Sports Phys. Ther..

[42] Kulas AS, Hortobágyi T, DeVita P (2012). Trunk position modulates anterior cruciate ligament forces and strains during a single-leg squat. Clin. Biomech..

[43] Mendiguchia J, Ford KR, Quatman CE, Alentorn-Geli E, Hewett TE (2011). Sex differences in proximal control of the knee joint. Sports Med. Auckl. NZ.

[44] Khuu A, Foch E, Lewis CL (2016). Not all single leg squats are equal: a biomechanical comparison of three variations. Int. J. Sports Phys. Ther..

[45] Khuu A, Lewis CL (2019). Position of the non-stance leg during the single leg squat affects females and males differently. Hum. Mov. Sci..

[46] Song C-Y, Huang H-Y, Chen S-C, Lin J-J, Chang AH (2015). Effects of femoral rotational taping on pain, lower extremity kinematics, and muscle activation in female patients with patellofemoral pain. J. Sci. Med. Sport.

[47] Scholtes SA, Salsich GB (2017). A dynamic valgus index that combines hip and knee angles: assessment of utility in females with patellofemoral pain. Int. J. Sports Phys. Ther..

[48] McKean M, Burkett BJ (2012). Does segment length influence the hip, knee and ankle coordination during the squat movement. J. Fit. Res..

[49] Wang J, Fu W (2019). Asymmetry between the dominant and non-dominant legs in the lower limb biomechanics during single-leg landings in females. Adv. Mech. Eng..

